# Comparison of human isogeneic Wharton’s jelly MSCs and iPSC-derived MSCs reveals differentiation-dependent metabolic responses to IFNG stimulation

**DOI:** 10.1038/s41419-019-1498-0

**Published:** 2019-03-20

**Authors:** Liani Devito, Michail E. Klontzas, Aleksandra Cvoro, Antonio Galleu, Marisa Simon, Carl Hobbs, Francesco Dazzi, Athanasios Mantalaris, Yacoub Khalaf, Dusko Ilic

**Affiliations:** 1 Department of Women and Children’s Health, King’s College London, Guy’s Hospital, London, UK; 2 Department of Chemical Engineering, Imperial College London, London, UK; 3 Genomic Medicine, Houston Methodist Research Institute, Houston, TX USA; 4 Department of Haemato-oncology, Rayne Institute, King’s College London, London, UK; 5 Histology Laboratory, Wolfson Centre for Age-Related Diseases, King’s College London, London, UK; 6 Present Address: Wallace H. Coulter Department of Biomedical Engineering, Georgia Institute of Technology, 950 Atlantic Drive, Engineering Biosciences Building, Rm 3016, Atlanta, GA 30332 USA

## Abstract

Variability among donors, non-standardized methods for isolation, and characterization contribute to mesenchymal stem/stromal cell (MSC) heterogeneity. Induced pluripotent stem cell (iPSCs)-derived MSCs would circumvent many of current issues and enable large-scale production of standardized cellular therapy. To explore differences between native MSCs (nMSCs) and iPSC-derived MSCs (iMSCs), we developed isogeneic lines from Wharton’s jelly (WJ) from the umbilical cords of two donors (#12 and #13) under xeno-free conditions. Next, we reprogrammed them into iPSCs (iPSC12 and iPSC13) and subsequently differentiated them back into iMSCs (iMSC12 and iMSC13) using two different protocols, which we named ARG and TEX. We assessed their differentiation capability, transcriptome, immunomodulatory potential, and interferon-γ (IFNG)-induced changes in metabolome. Our data demonstrated that although both differentiation protocols yield iMSCs similar to their parental nMSCs, there are substantial differences. The ARG protocol resulted in iMSCs with a strong immunomodulatory potential and lower plasticity and proliferation rate, whereas the TEX protocol raised iMSCs with a higher proliferation rate, better differentiation potential, though weak immunomodulatory response. Our data suggest that, following a careful selection and screening of donors, nMSCs from umbilical’s cord WJ can be easily reprogrammed into iPSCs, providing an unlimited source of material for differentiation into iMSCs. However, the differentiation protocol should be chosen depending on their clinical use.

## Introduction

Mesenchymal stromal/stem cells (MSCs) are a heterogenous population of fibroblastic cells that can be isolated from multiple tissues^1,2^. The plasticity, in vitro proliferation, immunomodulatory characteristics, and low immunogenicity of these cells made them an important instrument in the treatment of severe graft vs. host disease (GvHD)^3^. The number of clinical trials involving MSCs has been steadily increasing—from 218 clinical trials in 2012 till nearly 900 now. In spite of the rapidly growing body of literature and multiple clinical trials that have demonstrated the therapeutic benefits of MSC-mediated immune modulation variability among donors, non-standardized methods for their isolation and characterization produced inconsistent results from MSC-based cellular therapies^4,5^. The lack of standardization has been acknowledged by regulatory bodies and calls were made to improve characterization and refine selection criteria for establishing optimal classes of MSCs^6–9^.

To circumvent these issues, numerous groups, instead of working with primary MSCs, turned to human pluripotent stem cells using both human embryonic and induced pluripotent stem cells (hESC/iPSCs) as a source for derivation of MSC-like cells with potent therapeutic properties^10–21^. hESC/iPSC-derived MSCs could be derived under reproducible conditions and easily adapted to large-scale production of standardized cellular therapy. Indeed, quite recently, in May 2017, the first patient with steroid-refractory GvHD has been treated with hiPSC-derived MSCs in a clinical trial ongoing in the UK and Australia^22,23^.

However, to the best of our knowledge, only one study reported comparison of primary MSCs and hiPSC-derived MSCs in an isogeneic system—they reprogrammed bone marrow (BM) MSCs into hiPSC and then re-differentiated them back into MSC^24^. Comparison of DNA methylation between native MSC (nMSC) and hiPSC-derived MSC (iMSC) revealed that although iMSC are similar to nMSC, they do not entirely re-acquire immunomodulatory function in vitro nor do they achieve the original methylation patterns associated with tissue type and ageing.

We explored the isogeneic nMSC/iMSC approach and tested a different source of MSCs, different methods of reprogramming and multiple differentiation protocols. Instead of BM MSCs, we opted for MSCs isolated from Wharton’s jelly in umbilical cords (WJ MSC). WJ MSC are easily obtained, isolated, and expanded in vitro under defined xeno-free (dXF) conditions^25^. We also exploited genome non-integrating reprogramming approaches with Sendai virus (SeV)-based delivery instead of using traditional retroviral vector systems^26^. Finally, to differentiate iMSCs from iPSCs, we used two protocols: one described by Frobel et al.^24^ labeled ARG and one based on the experimental approach by Zhao et al.^14^ labeled TEX (Supporting Information Fig. S1).

## Methods and materials

### nMSC derivation and maintenance

The National Health Service Research Ethics Committee has approved the protocol for isolation of MSCs from perinatal tissues for cellular therapy (14/SW/0042). The collection of umbilical cords, derivation of MSCs, and their maintenance were described previously^25^.

### Reprogramming of WJ MSC

The native MSCs (nMSCs) from two donors, #12 and #13, were reprogrammed using CytoTune®-iPS Sendai Reprogramming kit. Reprogramming and detection of SeV expression in iPSC lines was described previously^26^. The modifications include a replacement of animal-based reagents with the xeno-free ones.

### Cell culture

iPSC, nMSC, and iMSC lines were expanded under xeno-free conditions in 5% O_2_, 5% CO_2_ at 37˚C as described previously^25^. In brief, nMSC lines that were derived and cultured under dXF conditions, on CELLstart (Thermo Fischer)-coated surface in StemGro® MSC Medium (Corning), were used for reprogramming into iPSC lines. iPSC lines were expanded on Vitronectin XF (StemCell Technologies)-coated surface in TeSR2 (StemCell Technologies). For comparative analyses in this study, nMSC and iMSC lines were propagated in α Minimum Essential Medium (αMEM, Thermo Fisher) supplemented with 10% human platelet lysate (hPL) (Cook Regentec). To avoid potential effect from different lots of hPL, the same lot has been used for culture of all lines in each of the experiments.

For passaging iPSC lines we used Accutase^TM^ (STEMCELL Technologies) and for nMSC and iMSC TripLE (Thermo Fisher).

To assess effects of interferon-γ (IFNG) on MSCs, recombinant human IFNG (Peprotech) was added to the media at 10 ng/ml and cells were cultured for 18 h prior to collection for RNA extraction

Acute myeloid leukemia cell line Katsumi, used as a CD34/CD45-positive control for flow cytometry, was cultured in RPMI-1640 medium supplemented with 20% fetal calf serum (FCS) at 37 °C, 5% CO2, 20% O2.

### Characterization of iPSC lines

Characterization of iPSC lines has been described in detail previously^26–29^. Once the lines are confirmed negative for SeV^26,27^, they are subjected to further characterization:

- Morphology—iPSC lines have to (i) propagate for > 3 passages, (ii) have well-defined colony edges, (iii) form a tightly packed colony, and (iv) have an absence of differentiated cells.
- Detection of pluripotency-associated markers—immunodetection of pluripotency-associated markers TRA-1-81, POU class 5 homeobox 1 (POU5F1)/octamer-binding transcription factor 4 (OCT4), TRA-1-60, and NANOG^27,28^.
- Determination of genomic stability—we use array-comparative genomic hybridization to determine genomic stability of the iPSC lines every three months of continuous culture^27,28^.
- Genotyping—amplification of polymorphic microsatellite markers is used for cell line identification at the molecular level^27,28^.
- Differentiation into three germ layers in vitro—iPSC colonies were left to differentiate without passage over a period of at least 3 weeks in Dulbecco’s modified Eagle’s medium supplemented with 10% FCS (Hyclone). The cells are then fixed and permeabilized in the dish. Markers representing the three germ layers, mesoderm, ectoderm, and endoderm, are detected with immunostaining^27,28^.
- Differentiation into three germ layers in vivo—2 × 10^6^ iPSC suspended in Matrigel were injected subcutaneously into flanks of NOD/SCID mice. The mice were killed 6–10 weeks later, depending on size of the tumor. Tumors are dissected and fixed in 4% paraformaldehyde. Tumor sections are stained with hematoxylin and eosin and analyze them under a light microscope. The presence of tissue-specific markers of all three germ layers has been confirmed by immunohistochemistry^27,28^.

### Characterization of nMSC and iMSC lines

Characterization of MSC lines has been described in detail previously^25,29^. In brief:

- Flow cytometry analysis and cell sorting—1 × 10^6^ cells were resuspended in 100 μL of ice-cold phosphate buffer saline (PBS) supplemented with 10% FBS, mixed with the appropriate primary antibody 1:50 and incubated in the dark for 15 min at room temperature. After washing with PBS, the cells were resuspended in 0.5 ml of cold PBS supplemented with 10% FCS. All antibodies used in flow cytometry and cell sorting were purchased from Miltenyi Biotec: phycoerythrin-conjugated anti-CD34, anti-CD73, and anti-mesenchymal stromal cell antigen-1 (MSCA-1), allophycocyanin-conjugated anti- CD56, anti-CD29 and anti-CD105, fluorescein-conjugated anti-CD44, anti-CD90, and anti-CD271, and Vio-Blue-conjugated anti-CD45. Titration was done for each of antibody used. Unstained cells were used as negative control. Fluorescence minus one was also performed to avoid any nonspecific binding. Expression of cell surface molecules was assessed using a FACS CANTO II (BD) and the data were analyzed using the DIVA software.
- Differentiation assays—the differentiation potential of nMSCs and iMSCs has been validated in three independent experiments using complete STEMPRO® adipogenesis, chondrogenesis or osteogenesis differentiating medium (Gibco®, Life Technologies) according to manufacturer’s protocol. Following fixation, the cellular lipids were detected cells with LipidTOX^TM^ Green Neutral Lipid Stain (Thermo Fisher) and nuclei with Hoechst 33342 (Thermo Fisher). The samples were mounted in Vectashield (Vector) and visualized using epifluorescence microscope (Nikon ECLIPSE 50i). Ca^2+^-containing cells in osteogenic cultures were stained with Alizarin red, whereas the presence of cartilage-related glycosaminoglycans in chondrogenic cultures was detected with Alcian blue staining.
- Real-time quantitative PCR (qPCR)—mRNA expression levels of osteogenic (*RUNX2*), chondrogenic (*COL11A1*), and adipogenic (*FABP4*) markers in all nMSC and iMSC lines were determined using the iScript cDNA Synthesis Kit (Bio-Rad) and SYBR Green Mastermix (Roche) in LightCycler 480 II (Roche). The primer sequences are listed in Supporting Information (Table S1).

### Differentiation of iPSC into iMSC

We generated iPSC-derived MSCs (iMSCs) using xeno-free modifications of two protocols. The first differentiation protocol, called ARG, was based on the protocol described by several groups^13,24,30^ and lasted 30 days. The iPSCs disaggregated into single cell suspension were seeded on Vitronectin XF-coated plates and cultured in αMEM (Thermo Fisher) supplemented with 10% hPL (Cook Regentec). Until Day 14 of differentiation, the medium also contained 2% B27 Supplement (Thermo Fisher) and the passaging was done in the presence of 10 µm Y27632 (STEMCELL Technologies).

The second protocol, called TEX, was based on the experimental approach by Zhao et al.^14^, who used SMAD 2/3 inhibitor to promote differentiation. In brief, the cells were plated on Vitroncetin XF-coated wells of six-well plates and cultured in TeSR2 supplemented with 10 μm SB-431542 (Sigma) for 25 days with continuous passaging (every 3–4 days) under the same culture conditions. During the next 20 days the cells were passaged on uncoated plastic. And the culture medium was then switched to standard MSC medium (αMEM supplemented with 10% hPL). All inhibitors were dissolved according to manufacturer’s recommendation.

At the end of the differentiation protocols, cells that were triple positive for CD73, CD90 and CD105 were sorted with a FACSAria II cell sorter (BD) and FACSDIVA software (BD) for acquisition and analysis, expanded and used for subsequent experiments.

### Transcriptomics

Total RNA was isolated from cells using the QIAgen RNeasy Mini kit (Qiagen). The RNA quality was assessed using Agilent RNA 6000 Nano Kit and the 2100 Bioanalyser (Agilent).

For the HumanHT-12 v4 Expression BeadChip (Illumina) assay RNA was isolated from cells with and without 18 h IFNG treatment. The samples were prepared using SuperScript III Reverse Transcriptase (Thermo Fisher) and TargetAmp Nano Labeling kit (Lucigen). Hybridization was performed according to the manufacturer’s instructions and run on the iScan system (Illumina). The data were analyzed by GenoSplice Technology using algorithm EASANA 2015.1.

The expression of the IFNG-target genes indoleamine 2,3-dioxygenase 1 (*IDO1*), guanylate-binding protein 4 (*GBP4*) and C-X-C motif chemokine ligand 11 (*CXCL11*) was assessed using the same RNA that was used for the BeadChip essay. Quantitative reverse transcription polymerase chain reaction (RT-qPCR) was performed using the StepOne System Instrument (Thermo Fisher) using a TaqMan® RNA-to-CT™ 1-Step Kit (Thermo Fisher) according to the manufacturer’s instructions. Data were collected and analyzed using the comparative threshold cycle method with hypoxanthine phosphoribosyltransferase 1 (*HPRT1*) as the reference gene. The primers are listed in Supporting Information (Table S1).

### Metabolomics

Metabolomics analysis was performed as previously reported^31–33^. In brief, culture medium was aspirated, cells were washed with PBS and metabolism quenched with ice-cold (−20˚C) methanol. Following the addition of high-performance liquid chromatography grade water (1:1 methanol:water), ribitol (1 mg/10^6^ cells, Sigma-Aldrich) and ^13^C-glucose (2 mg/10^6^ cells, Alfa Aesar) were used as internal standards based on the results of DNA quantification^34^. Samples were dried overnight under vacuum and the resulting dry polar metabolite mixtures were derivatized with the addition of 50 μL of methoxyamine hydrochloride (20 mg/mL in pyridine, Sigma-Aldrich) for 1.5 h, followed by 100 μL of *N*-methyl-trimethylsylyl-trifluoroacetamide for 6 h (Alfa Aesar)^35^. Each sample was run in quadruplicate injections in a Shimadzu QP2010 Ultra gas chromatography–mass spectrometry (GC-MS, Shimadzu).

### Statistics

qPCR data for markers of adipo-, chondro-, and osteogenesis were collected and analyzed using the comparative threshold cycle method with TATA-box binding protein (*TBP*) and glucuronidase β (*GUSB*) as reference genes. The mean ± standard deviation (SD) was calculated (*n* = 3 of each line), and statistical analysis was performed using the Prism curve-fitting program (GraphPad Prism, version 6.01). *P* values for expression of IFNG-target genes were determined with t-test and adjusted using Bonferroni correction. *p* ≤ 0.05 indicates statistically significant and *p* ≤ 0.001 highly significant values.

Transcriptomics data were analyzed by GenoSplice Technology using algorithm EASANA 2015.1. We used the R/Bioconductor software package limma that provides an integrated solution for differential expression analyses of data from gene expression experiments. To determine whether iMSC12 ARG or iMSC12 TEX is closer to paternal nMSC12, we used the R/Bioconductor software packages limma and hclust for Euclidean distance.

Metabolomics data preparation was accomplished according to Kanani et al.^36^ and bioinformatics analysis was performed with the use of R (R version 3.4.2, R studio 1.1.442) and TM4 MeV (version 4.9). Principal component analysis and hierarchical clustering with Manhattan distance metric were used for exploratory unsupervised multivariate analysis. Statistical analysis of multidimensional metabolomics data was performed with the use of Significance Analysis for Microarrays^37^ with the minimum significance threshold (*δ*) yielding an false discovery rate-median of 0%. Significant metabolites for each comparison were depicted on a reconstruction of the core MSCs’ metabolic network.

## Results

### Reprogramming and characterization of iPSC lines

We have previously demonstrated that WJ MSCs could be isolated successfully from the umbilical cord under dXF conditions^25^. The cells derived in this way retain all the characteristics of MSCs: the expression of typical MSC surface “markers”, differentiation potential into adipocytes, chondrocytes and osteocytes, and immunomodulatory activity. Therefore, to test our hypothesis that clinical grade iPSC-derived WJ MSCs can be generated from the beginning to the end under xeno-free conditions, we chose WJ MSCs derived and propagated under dXF conditions from two of seven donors for reprogramming into iPSC lines. The criterion for the selection of the donors was the size of the MSCA-1 + subpopulation (Supporting Information Table S2). MSCA-1 + cells are mesenchymal precursor cells and their expression is linked with an increased capacity of colony forming units > 100-fold in comparison with the MSCA-1- subpopulation^38–40^. WJ MSCs derived under dXF that had the highest number of MSCA-1 + cells were from donor 13 (86.1%), 12 (38.8%), and 14 (29.5%). To distinguish them from iPSC-derived MSC lines, we dubbed the primary native WJ MSCs as nMSCs. Therefore, from donor 12, we have nMSC12 and from donor 13, we have nMSC13. Both lines were from female donors (Supporting Information Fig. S2).

**Fig. 1:**
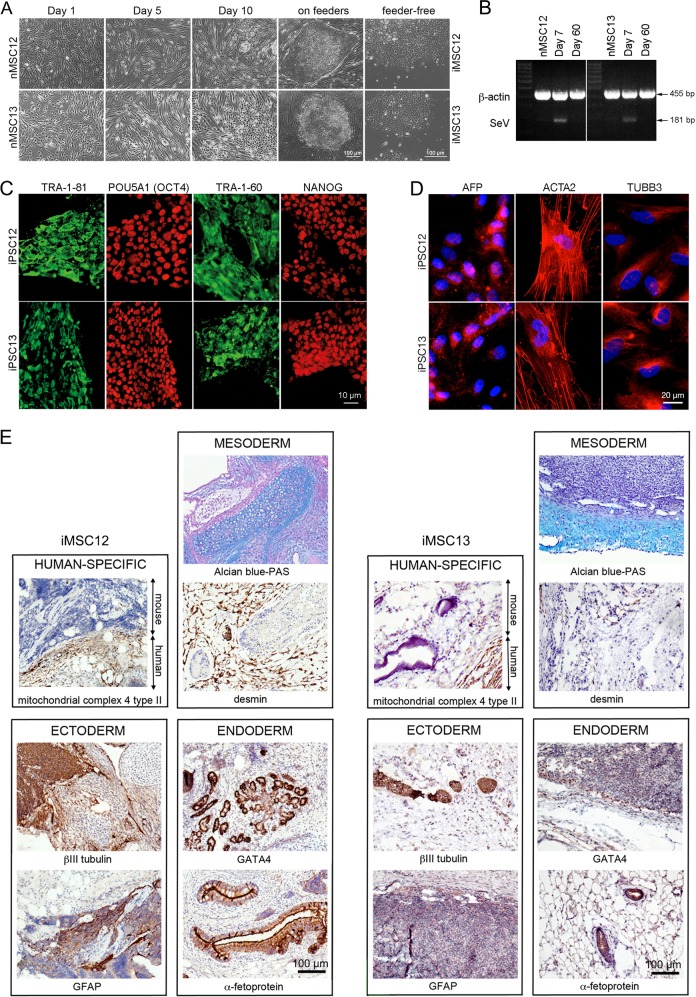
Reprograming nMSC into iPSC and their characterization. **a** Changes in morphology of nMSC12 during SeV-mediated reprogramming. **b** SeV was undetectable in iMSC12 and iMSC13 2 months after transduction. Original non-transduced native MSC lines (nMSC12 and nMSC13) were used as negative controls; 7 days post SeV transduction cells were used as positive controls (Day 7). At Day 60 in culture, the cells were negative for SeV expression. Β-actin (ACTB) PCR product is 455 bp, whereas SeV is 181 bp. MW: 100 bp ladder. **c** Both iPSC12 and iPSC13 express pluripotency-associated markers. iPSC colonies were positive for keratan sulfate antigens TRA-1-60 and TRA-1-81 (green) and transcription factors POU5A1 (OCT4) and NANOG (red). **d** iPSC12 and iPSC13 can spontaneously differentiate into derivatives of all three germ layers in vitro. Alpha-fetoprotein (AFP) demonstrated presence of endodermal derivatives, smooth muscle actin alpha 2 (ACTA2) of mesoderm and tubulin beta 3 class III (TUBB3) of ectoderm. **e** Spontaneous differentiation of iPSC12 and iPSC13 into all three germ layers in vivo is confirmed by detection of specific markers. All sections were stained with hematoxylin and eosin (H&E), whereas specific stains are brown (immunohistochemistry) or light blue (Alcian). Positive staining for mitochondrial complex IV type II confirms the human origin of the teratoma tissue. Germ layer markers: Alcian blue/periodic acid Schiff (PAS)-stained cartilage and DESMIN for mesoderm, TUBB3 and glial fibrillary acidic protein (GFAP) for ectoderm, and GATA-binding protein 4 (GATA4) and AFP for endoderm

Following reprogramming, one colony from each background was expanded further and adapted to feeder-free conditions (Fig. 1a)^41^. SeV was undetectable after 60 days in culture (Fig. 1b) and we could proceed with the characterization of the iPSC12 and iPSC13 lines. Both lines express pluripotency-associated markers, TRA-1-81, POU5F1 (OCT4), TRA-1-60, and NANOG (Fig. 1c) and can differentiate spontaneously into cells/tissues from all three germ layers in vitro (Fig. 1d) and in vivo (Fig. 1e).

### Differentiation of iMSCs and their characterization highlighted diversity of the MSC populations

**Fig. 2:**
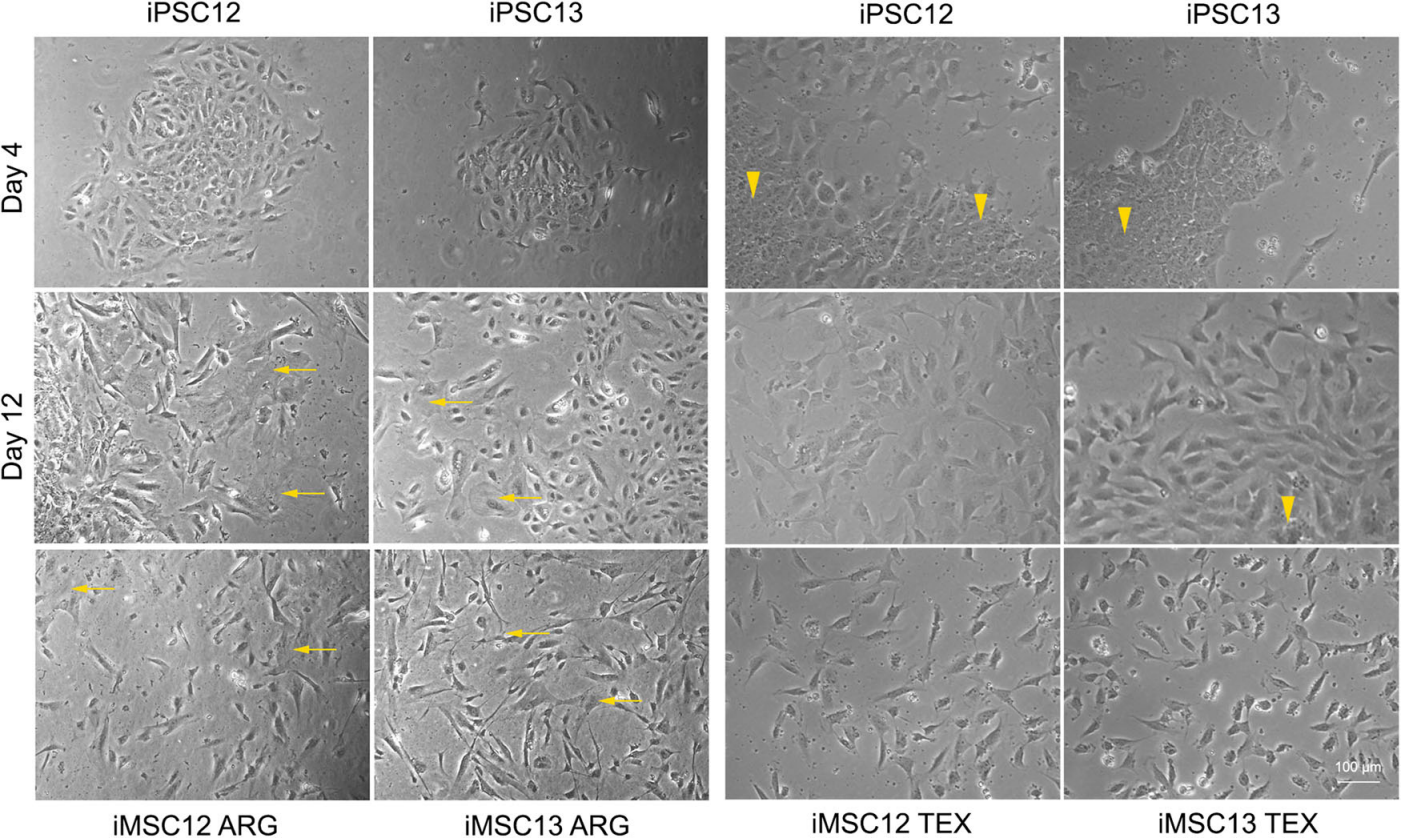
Change in cell morphology during differentiation of iPSCs into iMSC ARG. Top row (Day 4): tight cell–cell contacts, typical for densely packed pluripotent stem cell colonies, are completely lost in cells undergoing ARG differentiation protocol, whereas the fibroblast-like cells undergoing differentiation are mixed with seemingly intact pluripotent stem cell colonies (yellow arrowheads) in cell cultures undergoing TEX differentiation protocol. Middle row (Day 12): appearance of large flattened cells indicated cellular senescence-like features (yellow arrows) in cells undergoing ARG differentiation protocol. In contrast, all differentiating iPSC12 and iPSC13 have uniform fibroblast-like morphology. Bottom row: iMSCs growing on tissue culture-treated plastic. Senescent-like cells (yellow arrows) are present only in cell cultures undergoing ARG-differentiation protocol

To determine how close MSCs differentiated from pluripotent stem cells are to nMSC, we followed two different protocols: ARG^13,24,30^ and TEX^14^ with slight modifications to make them xeno-free. A difference between the two protocols was noted in cell morphology after 4 days (Fig. 2) and it persisted among CD73 + CD90 + CD105 + sorted cells cultured on tissue culture-treated plastic throughout the entire experimental work. Senescent-like cells were observed in both iPSC12 and 13 lines undergoing the ARG differentiation protocol as early as Day 12. They were not observed in either of two lines undergoing the TEX-differentiation protocol. This suggested that the iMSC12 TEX and iMSC13 TEX might maintain some of their stem cell-like characteristics and represent a type of MSC progenitor, whereas iMSC12 ARG, and iMSC13 ARG might be closer to primary MSC, which are a mixture of progenitors and the cells that lost stem cell-like characteristics and have more limited proliferation capacity.

**Fig. 3:**
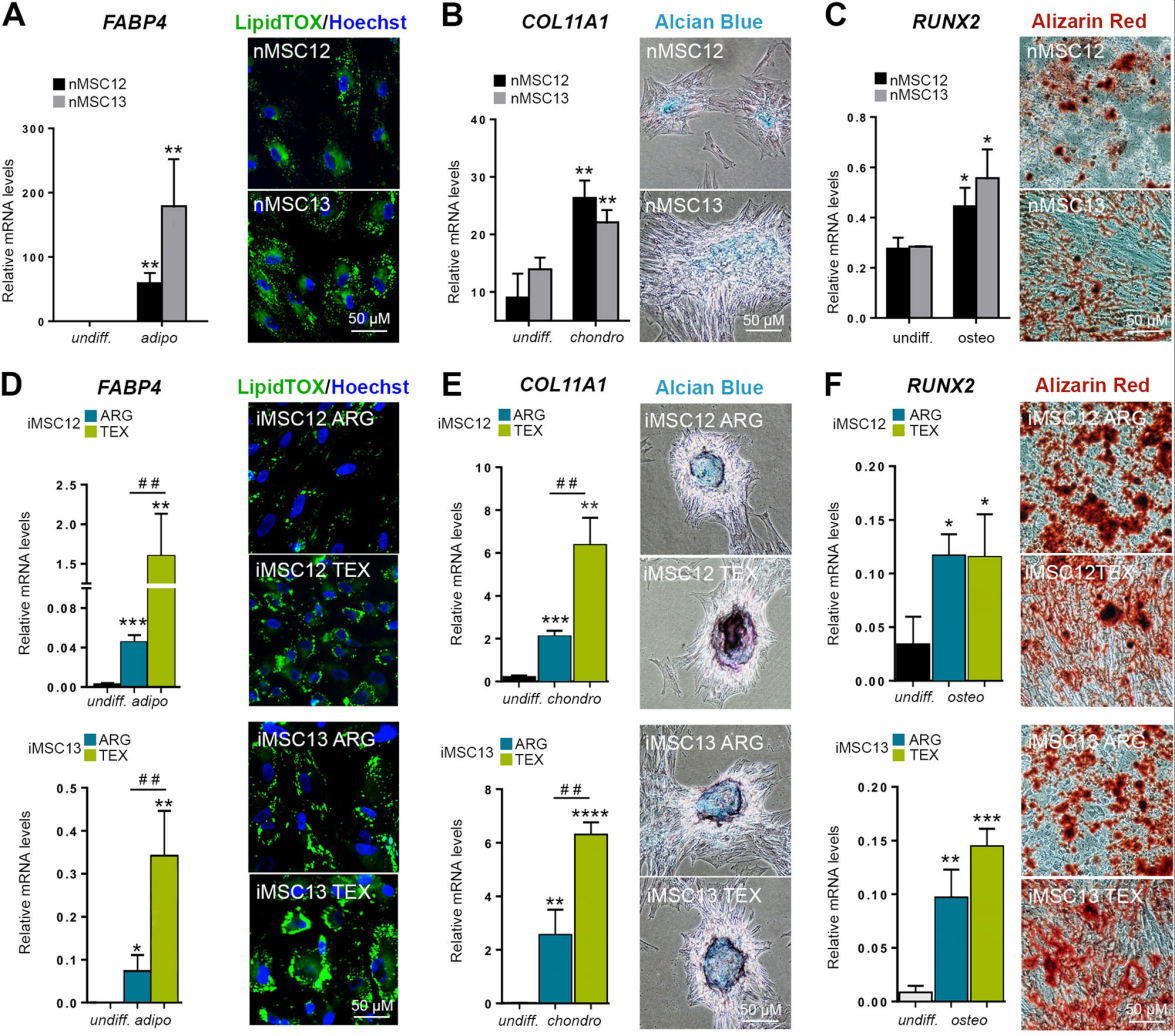
Differentiation potential of iMSCs. Differentiation potential. **a** nMSCs that were exposed to adipogenic differentiation medium showed accumulation lipid droplets as detected with LipidTOX stain and had a significantly higher (**p ≤ 0.01) expression of fatty acid binding protein 4 (*FABP4*) mRNA. No significant difference in *FABP4* mRNA levels was detected between nMSC12 and nMSC13. **b** nMSCs that were exposed to chondrogenic differentiation medium were positive for cartilage glycosaminoglycans as detected by Alcian Blue staining and had a significantly higher (**p ≤ 0.01) expression of collagen 11 (*COL11A1*) mRNA. No significant difference in *COL11A1* levels was detected between nMSC12 and nMSC13. **c** nMSCs that were exposed to osteogenic differentiation medium were full of mineral accumulations as detected by Alizarin Red staining and had a significantly higher (*p ≤ 0.05) expression of runt-related transcription factor 2 (*RUNX2*) mRNA. No significant difference in *RUNX2* levels was detected between nMSC12 and nMSC13. **d** Following adipogenic differentiation, all iMSCs lines showed accumulation of lipid droplets and significantly higher (***p ≤ 0.001, **p ≤ 0.01, *p ≤ 0.05) expression of *FABP4*. Both lines generated using the TEX protocol (iMSC12 TEX and iMSC13 TEX) had a significantly higher (^##^*p* ≤ 0.01) expression of *FAPB4* than the lines generated using the ARG protocol (iMSC12 ARG and iMSC13 ARG). **e** Following chondrogenic differentiation, all iMSC lines were positive for cartilage glycosaminoglycans as detected by Alcian Blue staining and had a significantly higher (****p ≤ 0.0001, ***p ≤ 0.001, **p ≤ 0.01) expression of *COL11A1*. Both lines generated using the TEX protocol (iMSC12 TEX and iMSC13 TEX) had a significantly higher (^##^p ≤ 0.01) expression of *COL11A1* than the lines generated using the ARG protocol (iMSC12 ARG and iMSC13 ARG). **f** Following osteogenic differentiation, all iMSC lines were full of mineral accumulation and had a significantly higher (***p ≤ 0.001, **p ≤ 0.01, *p ≤ 0.05) expression of *RUNX2*. There were no significant differences in *RUNX2* expression between the lines generated using the TEX protocol (iMSC12 TEX and iMSC13 TEX) and the lines generated using the ARG protocol (iMSC12 ARG and iMSC13 ARG) after culture under osteogenic conditions

Indeed, following iMSC differentiation into adipo-, chondro-, and osteogenic lineages, we found that the cells generated from iMSC using the TEX protocol had significantly higher expression of adipogenic and chondrogenic markers than those using ARG (Fig. 3). No significant difference between two protocols was observed only after culture under osteogenic conditions. These data are consistent with cellular morphology (Fig. 2), thus suggesting that lines of TEX origin might resemble progenitors more closely.

### TEX protocol yields iMSC with a transcriptomics profile closer to nMSC than ARG protocol

**Fig. 4:**
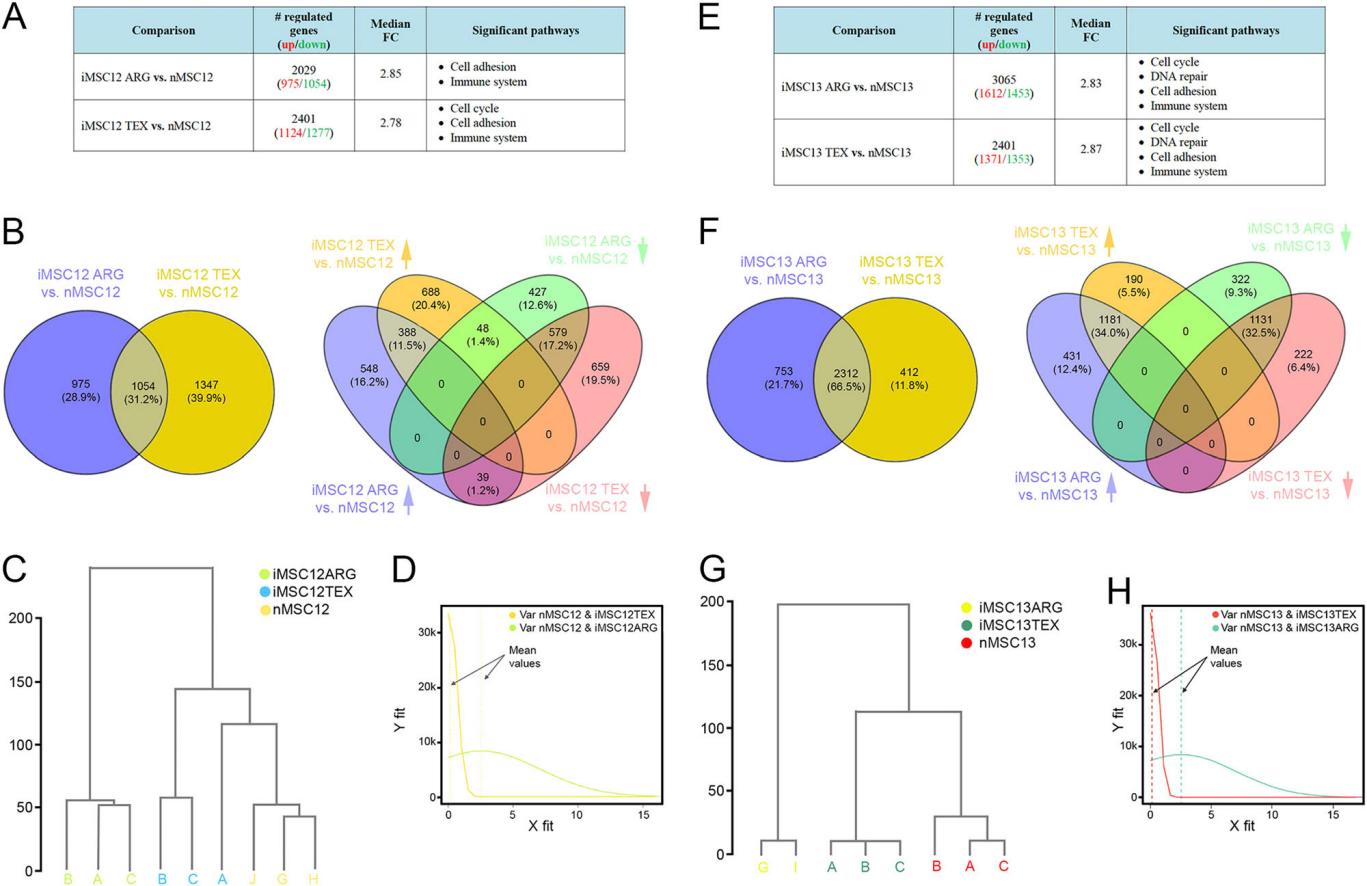
Transcriptome analyses from parental mMSC lines and iPSC-derived iMSC lines. **a** Differentially expressed genes in iMSC12. **b** Overlap between two comparisons (iMSC12 ARG vs. nMSC12 and iMSC12 TEX vs. nMSC12) shown as Venn diagrams. **c** Unsupervised clustering (Euclidian distance) of samples from donor 12 (nMSC12, iMSC12 ARG, and iMSC12 TEX). **d** Distribution variance and mean values for lines originated from donor 12. **e** Differentially expressed genes in iMSC13. **f** Overlap between two comparisons (iMSC13 ARG vs. nMSC13 and iMSC13 TEX vs. nMSC13) shown as Venn diagrams. **g** Unsupervised clustering (Euclidian distance) of samples from donor 13 (nMSC13, iMSC13 ARG, and iMSC13 TEX). **h** Distribution variance and mean values for lines originated from donor 13

Next, we compared transcriptome of iMSC12 and iMSC13 with their respective parental lines nMSC12 and nMSC13 (Fig. 4).

When compared with parental nMSC12, the number of differentially expressed genes is higher using TEX than the ARG protocol (2401 in iMSC12 TEX vs. 2029 in iMSC 12ARG; Fig. 4a). However, there were no significant differences between protocols in terms of fold-change median values (2.85 in iMSC12 ARG vs. nMSC12 and 2.78 in iMSC12 TEX and nMSC12). Approximately half of the genes with different regulation in iMSC12 ARG (1054 out of 2029 genes or 51.9%) are also differently regulated in iMSC12 TEX (1054 out of 2401 genes or 43.9%). Most of the common genes have the same up-/downregulation (Fig. 4b). To determine whether iMSC12 ARG or iMSC12 TEX are closer to paternal nMSC12, we used the R/Bioconductor software packages limma and hclust for Euclidean distance. Although there were more-regulated genes with TEX than ARG (2401 vs. 2029), based on the expression of all expressed genes, samples from the TEX protocol (iMSC12 TEX) were closer to nMSC12 than samples from the ARG protocol (iMSC12 ARG) (Fig. 4c). To explain this difference, we checked the variance distribution between iMSC12 ARG/nMSC12 and iMSC12 TEX/nMSC12 (Fig. 4d). The variances were higher between gene expression from the iMSC12 ARG/nMSC12 groups than between gene expression from the iMSC12 TEX/nMSC12 groups. Unsupervised clustering and variance analysis showed also that iMSC12 TEX cells are closer to nMSC12 than iMSC12 ARG.

Using the same approach, we then determined the differentially expressed genes and their associated pathways between iMSC13 TEX and nMSC13 and between iMSC13 ARG and nMSC13. We found that iMSC13 from both protocols (TEX and ARG) have a high number of differentially expressed genes (Fig. 4e). However, in this case, the number of differentially expressed genes was higher using ARG than TEX (3065 vs. 2724). Similarly, there were no significant differences between protocols in terms of fold-change median values (2.83 in iMSC13 ARG vs. nMSC13 and 2.87 in iMSC13 TEX and nMSC13). The majority of genes with different regulation in iMSC13 TEX (2312 out of 2724 genes or 84.9%) are also differently regulated in iMSC13 ARG (2312 out of 3065 genes or 75.4%). Most of the common differently regulated genes have the same up-/downregulation (Fig. 4f). Based on the expression of all expressed genes, samples from the TEX protocol (iMSC13 TEX) were again closer to nMSC13 than samples from the ARG protocol (iMSC13 ARG) (Fig. 4g). The variances were higher between gene expression from the iMSC13 ARG/nMSC13 groups than between gene expression from the iMSC13 TEX/nMSC13 groups (Fig. 4h). Unsupervised clustering and variance analysis together show that iMSC13 TEX cells are closer to nMSC13 than iMSC13 ARG.

The sum of the data indicates that the TEX protocol produces iMSCs closer to nMSCs.

### ARG protocol yields iMSCs that respond to IFNG similar to nMSC

**Fig. 5:**
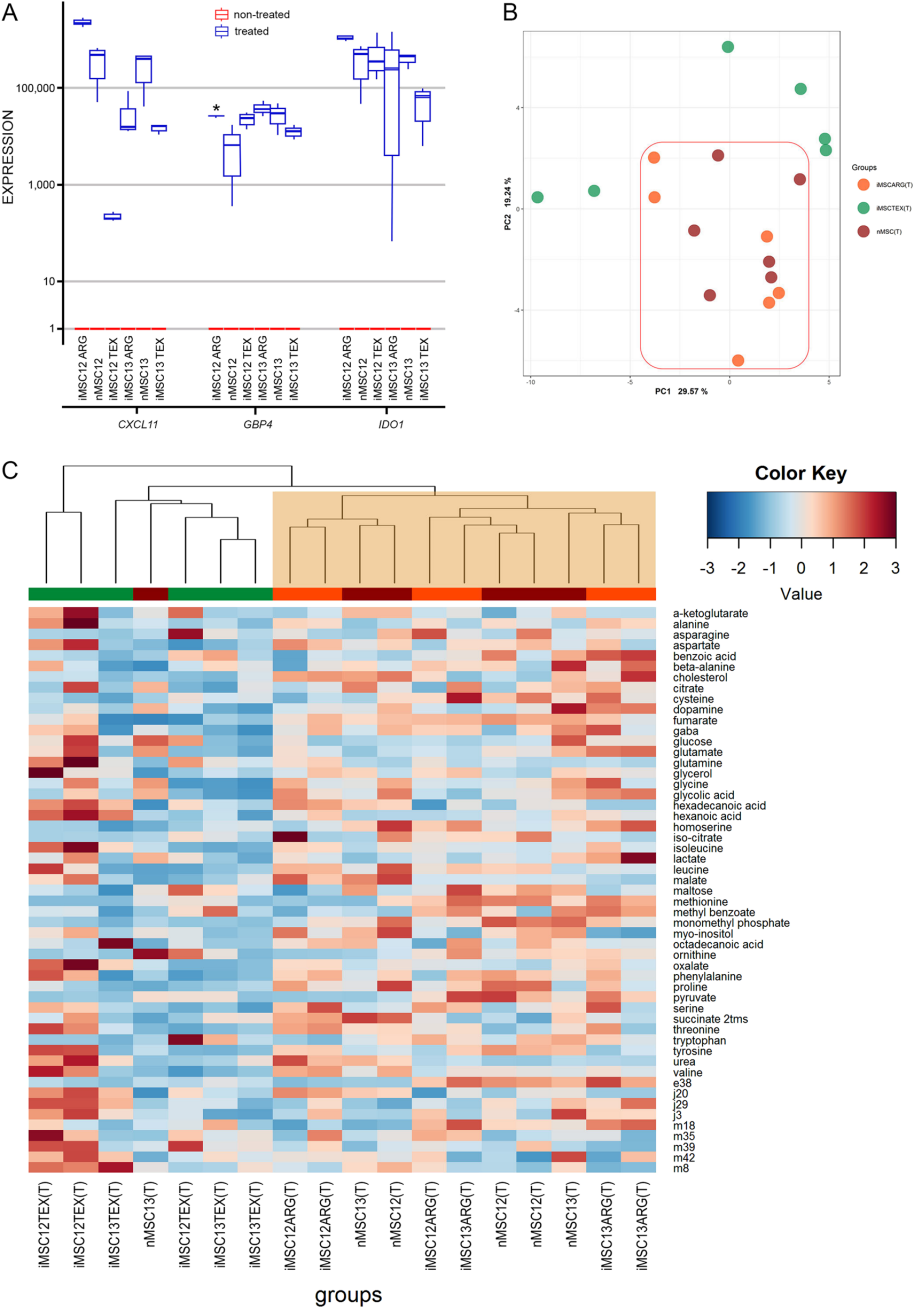
Different metabolomic response of iMSC to IFNG treatment. **a** qPCR analysis confirmed that IFNG treatment induced expression of *CXCL11*, *GBP4*, and *IDO1* in all our six iMSC lines (*n* = 3 for each of non-treated and IFNG-treated samples). Red lines represent values in non-treated cells. Only two treated individual lines significantly differed in one gene expression. p values were determined with *t* test and adjusted using Bonferroni correction. *, *p* ≤ 0.05. **b**, **c** Upon IFNG treatment (T), PCA (B) and hierarchical clustering with Manhattan distance metric (C) of metabolomics showed increased global metabolic activity only in nMSC (T) and iMSC ARG (T), but not in iMSC TEX (T) lines

As MSCs can exert potent immunosuppressive and immunoregulatory effects^5,42,43^, we assessed their response to IFNG treatment (T). To verify that IFNG treatment induced the expected signaling response, we compared mRNA levels of *IDO1*, *GBP4*, and *CXCL11* between untreated and treated cells by qPCR. These three genes have been used extensively as markers of IFNG response^44–46^. Both nMSC and all iMSC lines displayed a similar increase in expression of these three markers (Fig. 5a).

Next, we performed metabolomics analysis of nMSCs and iMSCs to evaluate the changes in metabolic physiology in response to IFNG treatment and identify potential protocol-specific (ARG or TEX) differences. Treatment with IFNG was found to globally increase the metabolic activity of both nMSCs and iMSC ARG as shown by principal component analysis (Fig. 5b) and hierarchical clustering, where the profiles of iMSC ARG (T) cluster together with the profiles of nMSC(T) despite the variability in metabolic physiology before IFNG treatment (Fig. 5c). Clustering between iMSC ARG(T) and nMSC(T) denotes the higher metabolic similarity upon IFNG stimulation between cells derived with ARG and native cells.

**Fig. 6:**
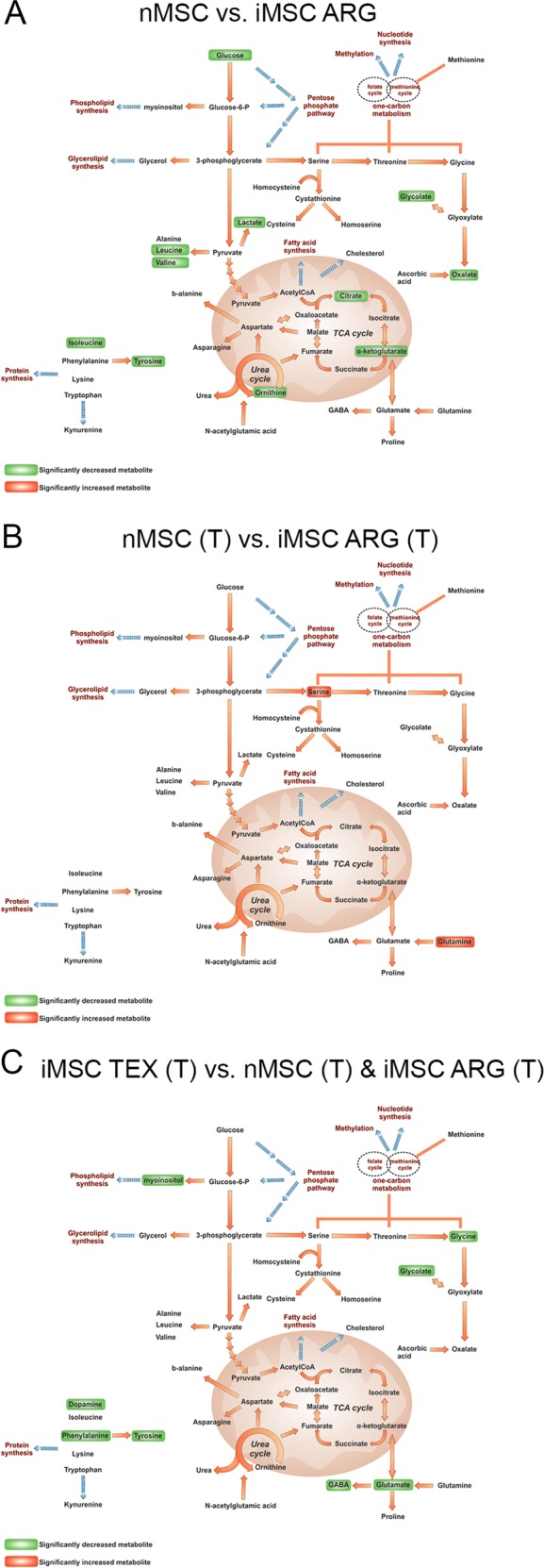
On-network representation of significant metabolic changes. **a** Comparison of nMSCs vs. iMSC ARG, showed that iMSC ARG had a lower glycolytic and glutaminolytic activity, lower levels of TCA and urea cycle metabolites and decreased amino-acid pools compared with nMSCs. **b** IFNG stimulation diminished the differences in metabolic phenotype between nMSC (T) and iMSC ARG (T) seen in **a**. The only difference are increased levels of glutamine and serine in iMSC ARG (T). **c** INFG treatment did not increase metabolic activity of iMSC TEX (T). Glutaminolysis and the levels of biosynthetic precursors (amino acids and lipid precursors such as myoinositol) was significantly lower in comparison with both nMSC (T) and iMSC ARG (T). (T), IFNG-treated cells

Direct on-network significance comparison of nMSCs vs. iMSC ARG, showed that iMSC ARG had a lower glycolytic and glutaminolytic activity, lower levels of tricarboxylic acid cycle (TCA) and urea cycle metabolites and decreased amino-acid pools compared with their native equivalents (Fig. 6a). Upon IFNG stimulation, the differences in metabolic phenotype between nMSC (T) and iMSC ARG (T) were limited to increased levels of glutamine and serine in iMSC ARG (T) (Fig. 6b). iMSC TEX did not respond to IFN treatment in a similar way to iMSC ARG. iMSC TEX were found to possess a highly variable metabolic physiology. In iMSC TEX (T), glutaminolysis and the levels of biosynthetic precursors (amino acids and lipid precursors such as myoinositol) were significantly lower compared to both other groups (Fig. 6c).

## Discussion

We used a model system of isogeneic primary WJ nMSCs and iPSC-derived iMSCs to explore differences between native WJ MSCs, nMSCs, and iMSCs, originating from these nMSCs. A similar system has been reported by Frobel et al.^24^. They found differences in epigenetic signature, particularly in patterns associated with tissue type and ageing. They also reported incomplete reacquisition of immunomodulatory properties, which prompted us to analyze response to the well characterized pro-inflammatory cytokine IFNG, secreted by cells of both the innate and adaptive immune systems.

We employed two different differentiation protocols: (1) ARG was very similar to the protocol described by Frobel et al.^24^ and (2) TEX was similar to Zhao et al.^14^. We found that two iMSC ARG lines we analyzed responded similarly to IFNG treatment as parental nMSC, indicating that their immunomodulatory properties might be preserved. There are several differences between our isogeneic system and the system reported by Frobel et al.^24^, which might contribute to the different findings. Interestingly, the data from iMSC lines differentiated using TEX, had attenuated response to IFNG stimulation. We do not know for sure why TEX retained more stem cell-like phenotype improved the differentiation potential, whereas ARG preserved immunomodulation better. We can only speculate that is due to exposure of cells to transforming growth factor beta (TGFB) signaling inhibitor (10 μm SB-431542) in TEX protocol. TGFB family signaling is implicated in regulation of stemness, however, the effects are diverse and depend on the cell types as well as cell physiology and environmental factors^47^.

The concept that multiple connective tissues have common progenitor stem cell retained throughout adulthood at various locations in the body has been debated within the community, though not proven yet, and the concept remains ambiguous^9^. BM MSCs that were used for reprogramming and derivation of iMSC are isolated from adults and from a different tissue type then ours. Although WJ MSCs share common features with BM MSCs and MSC from other adult tissues, such as being positive for CD44, CD73, CD90, CD105, α-smooth muscle actin, and vimentin^48,49^, they still might be a distinct population. Moreover, the term WJ MSCs is often extended to all umbilical cord stromal cells even though they are residing in different distinct zones of the WJ matrix—the sub-amnion, WJ per se, and peri-vascular zone^50^.

Defects in immunomodulatory response of iMSCs found by Frobel et al.^24^ could be also an effect of difference in culture conditions. Most cells in culture are grown in an atmosphere of 5% CO_2_, 95% air, which represents an incubator oxygen concentration of 20% (160 mm Hg). Such tissue culture conditions are strongly oxidizing. For example, oxygen gradients within the human BM are in a range from below 1% in hypoxic niches to 6% in the sinusoidal cavity^51^. It has been demonstrated that such low oxygen levels are essential for maintenance of cell’s stemness-related properties in all MSCs including adipose-derived^52^ and WJ MSC^25,53–56^. To mimic physiological conditions as much as possible in vitro, we cultured the cells in all stages at 5% O_2_. In addition, following modification of the original protocol^24,30^ introduced by Luzzani et al.^13^, we also supplemented the iPSC-differentiation medium with B27 Supplement, which supports cell survival with a defined spectrum of biological antioxidants^57,58^.

Although we used different reprogramming techniques (genome-integrating retrovirus vs. genome non-integrating SeV), we do not believe that they are behind differences in immunomodulatory effects we observed, and those published by Frobel et al.^24^. Three recent studies have compared the genomic features of iPSCs from between 100 and 300 individuals, and the iPSC lines have shown to be heterogeneous, raising questions about the suitability of these lines for genetic studies^59–61^. These studies were able to show that the genetic background of each individual has a superior influence on variation in the iPSC lines compared with other non-genetic factors, including copy number status, culture conditions, passage, and gender. Therefore, it is hypothesized that the majority of iPSC heterogeneity is driven by inherent genetic variation between individuals, rather than by any variation in culture conditions and duration, or in the reprogramming method used. All three reports showed that a great amount of genomic variations between the iPSC lines affected the genes involved in stem cell maintenance and the differentiation efficiency of the iPSCs. Our iMSC lines from both groups, TEX and ARG, showed individual differences in gene expression that could be attributed to the genetic background to each parental cell.

Stimulation of nMSCs with IFNG lead to increased metabolism (Supporting Information Fig. S3) and diminished metabolic differences between nMSC and iMSC ARG (Fig. 6a, b). However, we could not detect such change in overall metabolism of iMSC TEX after IFNG treatment (Fig. 5b, c), even though they responded to IFNG with increased synthesis of downstream effectors such as *IDO1*, *GBP4,* and *CXCL11* (Fig. 5a). Although it is very little known about metabolic changes in MSC when they are exposed to inflammatory cytokines such as IFNG, and how that would corelate with their immunomodulatory capabilities, it is possible to draw a parallel with metabolic changes during macrophage or T-cell activation^62,63^. Increase in both energy production and biomass such as amino acids, lipids, and nucleic acids, is necessary to provide cellular building blocks to mobilize all the resources required to fully activate and use their host-defense mechanisms. Since such metabolic change was not detected in iMSC TEX cells, it is likely that they may not be capable of exerting expected immunomodulatory response in an inflammatory environment. Despite the similarity between iMSC ARG (T) and nMSC(T), metabolism of iMSC ARG was different to nMSC. This signifies the hypothesis that IFNG has a potent effect on stem cell physiology, leading cells to a specific metabolic state, despite the metabolic differences prior to stimulation.

The current study has several limitations, i.e., testing of immunomodulatory properties has not been done in animal model setting; nonetheless, it still demonstrates the importance of validating clinically relevant characteristics of iMSC, especially in an isogeneic system. The results of our mass spectrometry and metabolomics analyses presented provide the required evidence and the animal data would not necessarily add much more value. We have also illustrated how metabolomics can be used as a reliable tool for validating cellular response to pro-inflammatory stimuli. In spite of the increase in transcription of marker genes downstream of IFNG receptor activation, the signal did not reach the threshold required for setting in motion an immunomodulatory mechanism.

## Supplementary information

Supplemental Material

## References

[1] Haniffa MA, Collin MP, Buckley CD, Dazzi F (2009). Mesenchymal stem cells: the fibroblasts’ new clothes?. Haematologica.

[2] Haniffa MA (2007). Adult human fibroblasts are potent immunoregulatory cells and functionally equivalent to mesenchymal stem cells. J. Immunol..

[3] Nombela-Arrieta C, Ritz J, Silberstein LE (2011). The elusive nature and function of mesenchymal stem cells. Nat. Rev. Mol. Cell Biol..

[4] Trento C (2018). Manufacturing mesenchymal stromal cells for the treatment of graft-versus-host disease: a survey among centers affiliated with the European Society for Blood and Marrow Transplantation. Biol. Blood. Marrow Transplant..

[5] Wang Y, Chen X, Cao W, Shi Y (2014). Plasticity of mesenchymal stem cells in immunomodulation: pathological and therapeutic implications. Nat. Immunol..

[6] Samsonraj RM (2017). Multifaceted characterization of human mesenchymal stem cells for use in regenerative medicine. Stem. Cells Transl. Med..

[7] Galipeau J (2016). International Society for Cellular Therapy perspective on immune functional assays for mesenchymal stromal cells as potency release criterion for advanced phase clinical trials. Cytotherapy.

[8] Viswanathan S (2014). Soliciting strategies for developing cell-based reference materials to advance mesenchymal stromal cell research and clinical translation. Stem. Cells Dev..

[9] Bianco P (2013). The meaning, the sense and the significance: translating the science of mesenchymal stem cells into medicine. Nat. Med..

[10] Hawkins KE (2018). Embryonic stem cell-derived mesenchymal stem cells (MSCs) Have a superior neuroprotective capacity over fetal MSCs in the hypoxic-ischemic mouse brain. Stem. Cells Transl. Med..

[11] Sheyn D (2016). Human induced pluripotent stem cells differentiate into functional mesenchymal stem cells and repair bone defects. Stem. Cells Transl. Med..

[12] Koch JM, D’Souza SS, Schwahn DJ, Dixon I, Hacker TA (2016). Mesenchymoangioblast-derived mesenchymal stromal cells inhibit cell damage, tissue damage and improve peripheral blood flow following hindlimb ischemic injury in mice. Cytotherapy.

[13] Luzzani C (2015). A therapy-grade protocol for differentiation of pluripotent stem cells into mesenchymal stem cells using platelet lysate as supplement. Stem. Cell Res. Ther..

[14] Zhao Q (2015). MSCs derived from iPSCs with a modified protocol are tumor-tropic but have much less potential to promote tumors than bone marrow MSCs. Proc. Natl Acad. Sci. USA.

[15] Kimbrel EA (2014). Mesenchymal stem cell population derived from human pluripotent stem cells displays potent immunomodulatory and therapeutic properties. Stem. Cells Dev..

[16] Guzzo RM, Gibson J, Xu RH, Lee FY, Drissi H (2013). Efficient differentiation of human iPSC-derived mesenchymal stem cells to chondroprogenitor cells. J. Cell Biochem..

[17] Vodyanik MA (2010). A mesoderm-derived precursor for mesenchymal stem and endothelial cells. Cell Stem. Cell.

[18] Boyd NL, Robbins KR, Dhara SK, West FD, Stice SL (2009). Human embryonic stem cell-derived mesoderm-like epithelium transitions to mesenchymal progenitor cells. Tissue Eng. Part. A..

[19] Karlsson C (2009). Human embryonic stem cell-derived mesenchymal progenitors - potential in regenerative medicine. Stem. Cell Res..

[20] Lian Q (2007). Derivation of clinically compliant MSCs from CD105 + , CD24- differentiated human ESCs. Stem. Cells.

[21] Barberi T, Willis LM, Socci ND, Studer L (2005). Derivation of multipotent mesenchymal precursors from human embryonic stem cells. PLoS. Med..

[22] King, D. First human allogeneic clinical trial commences iPSC-derived mesenchymal stem cells. 2017. https://cellculturedish.com/2017/07/first-human-allogeneic-clinical-trial-commences-ipsc-derived-mesenchymal-stem-cells/ Accessed 19 Aug 2018.

[23] Clinicaltrials.gov https://clinicaltrials.gov/ct2/show/NCT02923375 Accessed 19 Aug 2018.

[24] Frobel J (2014). Epigenetic rejuvenation of mesenchymal stromal cells derived from induced pluripotent stem cells. Stem Cell Rep..

[25] Devito L (2014). Wharton’s jelly mesenchymal stromal/stem cells derived under chemically defined animal product-free low oxygen conditions are rich in MSCA-1( + ) subpopulation. Regen. Med..

[26] Miere C, Devito L, Ilic D (2016). Sendai virus-based reprogramming of mesenchymal stromal/stem cells from umbilical cord wharton’s jelly into induced pluripotent stem cells. Methods Mol. Biol..

[27] Devito L (2018). Induced pluripotent stem cell line from an atopic dermatitis patient heterozygous for c.2282del4 mutation in filaggrin: KCLi001-A. Stem. Cell Res..

[28] Petrova A (2014). 3D In vitro model of a functional epidermal permeability barrier from human embryonic stem cells and induced pluripotent stem cells. Stem. Cell Rep..

[29] Badraiq H (2017). Effects of maternal obesity on Wharton’s Jelly mesenchymal stromal cells. Sci. Rep..

[30] Shao K (2013). Induced pluripotent mesenchymal stromal cell clones retain donor-derived differences in DNA methylation profiles. Mol. Ther..

[31] Klontzas ME, Vernardis SI, Heliotis M, Tsiridis E, Mantalaris A (2017). Metabolomics analysis of the osteogenic differentiation of umbilical cord blood mesenchymal stem cells reveals differential sensitivity to osteogenic agents. Stem. Cells Dev..

[32] Vernardis SI, Terzoudis K, Panoskaltsis N, Mantalaris A (2017). Human embryonic and induced pluripotent stem cells maintain phenotype but alter their metabolism after exposure to ROCK inhibitor. Sci. Rep..

[33] Vernardis SI, Goudar CT, Klapa MI (2013). Metabolic profiling reveals that time related physiological changes in mammalian cell perfusion cultures are bioreactor scale independent. Metab. Eng..

[34] Silva LP (2013). Measurement of DNA concentration as a normalization strategy for metabolomic data from adherent cell lines. Anal. Chem..

[35] Kanani H, Chrysanthopoulos PK, Klapa MI (2008). Standardizing GC-MS metabolomics. J. Chromatogr. B. Anal. Technol. Biomed. Life. Sci..

[36] Kanani HH, Klapa MI (2007). Data correction strategy for metabolomics analysis using gas chromatography-mass spectrometry. Metab. Eng..

[37] Tusher VG, Tibshirani R, Chu G (2001). Significance analysis of microarrays applied to the ionizing radiation response. Proc. Natl Acad. Sci. USA.

[38] Sobiesiak M (2010). The mesenchymal stem cell antigen MSCA-1 is identical to tissue non-specific alkaline phosphatase. Stem. Cells Dev..

[39] Bühring HJ (2019). Phenotypic characterization of distinct human bone marrow-derived MSC subsets. Ann. N. Y. Acad. Sci..

[40] Gronthos S (2007). A novel monoclonal antibody (STRO-3) identifies an isoform of tissue nonspecific alkaline phosphatase expressed by multipotent bone marrow stromal stem cells. Stem. Cells Dev..

[41] Stephenson E (2012). Derivation and propagation of human embryonic stem cell lines from frozen embryos in an animal product-free environment. Nat. Protoc..

[42] Ma, S., et al. Immunobiology of mesenchymal stem cells. *Cell Death Differ.* **21**, 216–225. 10.1038/cdd.2013.158 PMC3890955 24185619

[43] Marigo I, Dazzi F (2011). The immunomodulatory properties of mesenchymal stem cells. Semin. Immunopathol..

[44] Krampera M (2006). Role for interferon-gamma in the immunomodulatory activity of human bone marrow mesenchymal stem cells. Stem. Cells.

[45] Vestal DJ (2005). The guanylate-binding proteins (GBPs): proinflammatory cytokine-induced members of the dynamin superfamily with unique GTPase activity. J. Interferon Cytokine Res..

[46] Tensen CP (1999). Genomic organization, sequence and transcriptional regulation of the human CXCL 11(1) gene. Biochim. Biophys. Acta.

[47] Sakaki-Yumoto M, Katsuno Y, Derynck R (2013). TGF-β family signaling in stem cells. Biochim. Biophys. Acta.

[48] Huang YC, Parolini O, La Rocca G, Deng L (2012). Umbilical cord versus bone marrow derived mesenchymal stromal cells. Stem. Cells Dev..

[49] De Kock J (2012). Mesoderm-derived stem cells: the link between the transcriptome and their differentiation potential. Stem. Cells Dev..

[50] Corrao S (2013). Umbilical cord revisited: from Wharton’s jelly myofibroblasts to mesenchymal stem cells. Histol. Histopathol..

[51] Eliasson P, Jonsson JI (2010). The hematopoietic stem cell niche: low in oxygen but a nice place to be. J. Cell Physiol..

[52] Yang S (2012). Defined xenogeneic-free and hypoxic environment provides superior conditions for long-term expansion of human adipose-derived stem cells. Tissue Eng. Part. C. Methods.

[53] Drela K (2014). Low oxygen atmosphere facilitates proliferation and maintains undifferentiated state of umbilical cord mesenchymal stem cells in an hypoxia inducible factor-dependent manner. Cytotherapy.

[54] López L, Seshareddy K, Trevino E, Cox J, Weiss ML (2011). Evaluating the impact of oxygen concentration and plating density on human Wharton’s jelly-derived mesenchymal stromal cells. Open Tissue Eng. Regen. Med. J..

[55] Lavrentieva A, Majore I, Kasper C, Hass R (2010). Effects of hypoxic culture conditions on umbilical cordderived human mesenchymal stem cells. Cell Commun. Signal..

[56] Nekanti U, Dastidar S, Venugopal P, Totey S, Ta M (2010). Increased proliferation and analysis of differential gene expression in human Wharton’s jelly-derived mesenchymal stromal cells under hypoxia. Int. J. Biol. Sci..

[57] Brewer GJ, Torricelli JR, Evege EK, Price PJ (1993). Optimized survival of hippocampal neurons in B27-supplemented Neurobasal, a new serum-free medium combination. J. Neurosci. Res..

[58] Brewer GJ, Cotman CW (1989). Survival and growth of hippocampal neurons in defined medium at low density: advantages of a sandwich culture technique or low oxygen. Brain Res..

[59] Carcamo-Orive I (2017). Analysis of transcriptional variability in a large human iPSC library reveals genetic and non-genetic determinants of heterogeneity. Cell Stem. Cell.

[60] DeBoever C (2017). Large-scale profiling reveals the influence of genetic variation on gene expression in human induced pluripotent stem cells. Cell Stem. Cell.

[61] Kilpinen H (2017). Common genetic variation drives molecular heterogeneity in human iPSCs. Nature.

[62] Domblides C, Lartigue L, Faustin B (2018). Metabolic stress in the immune function of T cells, macrophages and dendritic cells. Cells.

[63] Zhu L, Zhao Q, Yang T, Ding W, Zhao Y (2015). Cellular metabolism and macrophage functional polarization. Int. Rev. Immunol..

