# Supplementary material for: Comparison of human isogeneic Wharton’s jelly MSCs and iPSC-derived MSCs reveals differentiation-dependent metabolic responses to IFNG stimulation
Source: Cell Death Dis. 2019 Mar 20;10(4):277. doi: 10.1038/s41419-019-1498-0 (PMC6426992; doi:10.1038/s41419-019-1498-0)
Supplement: Supplementary file 1 — Supplemental Material [file 41419_2019_1498_MOESM1_ESM.docx]

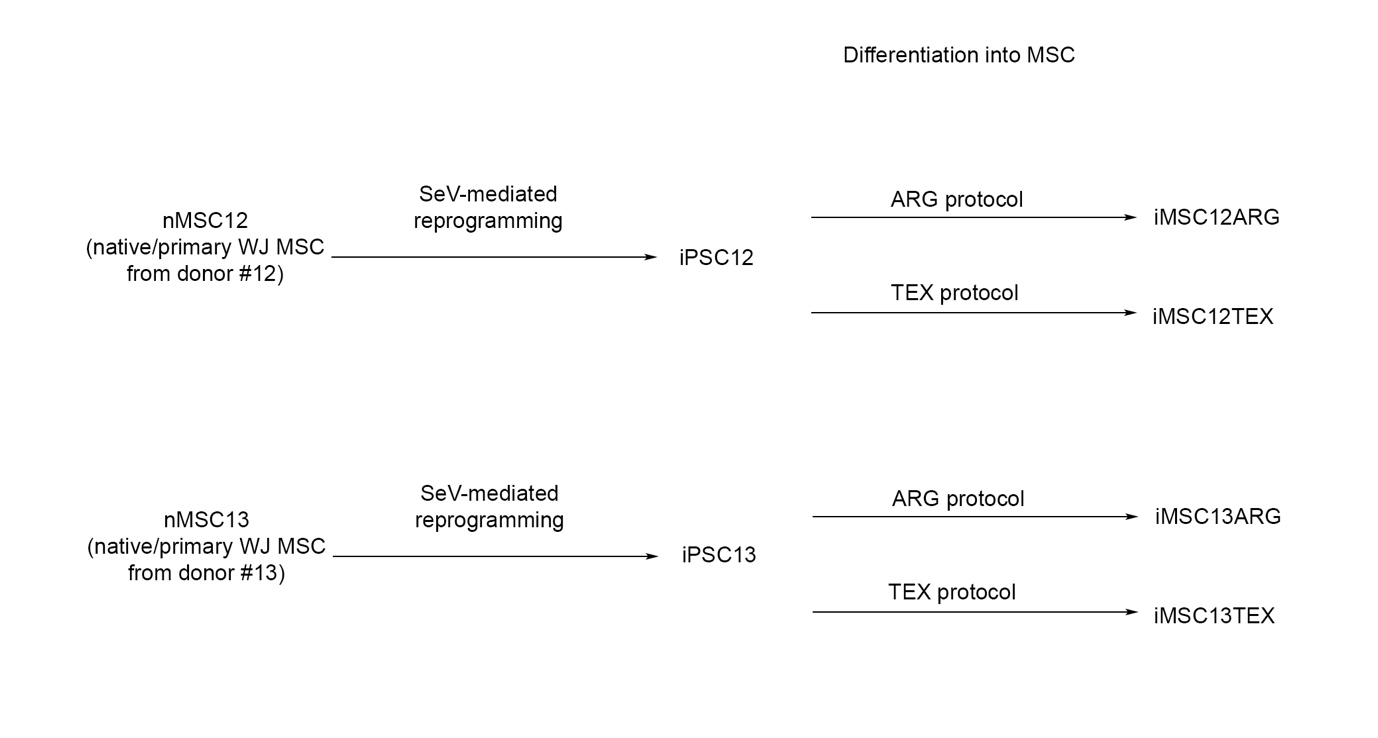


**Figure S1.** Schematic diagram of experimental strategy.


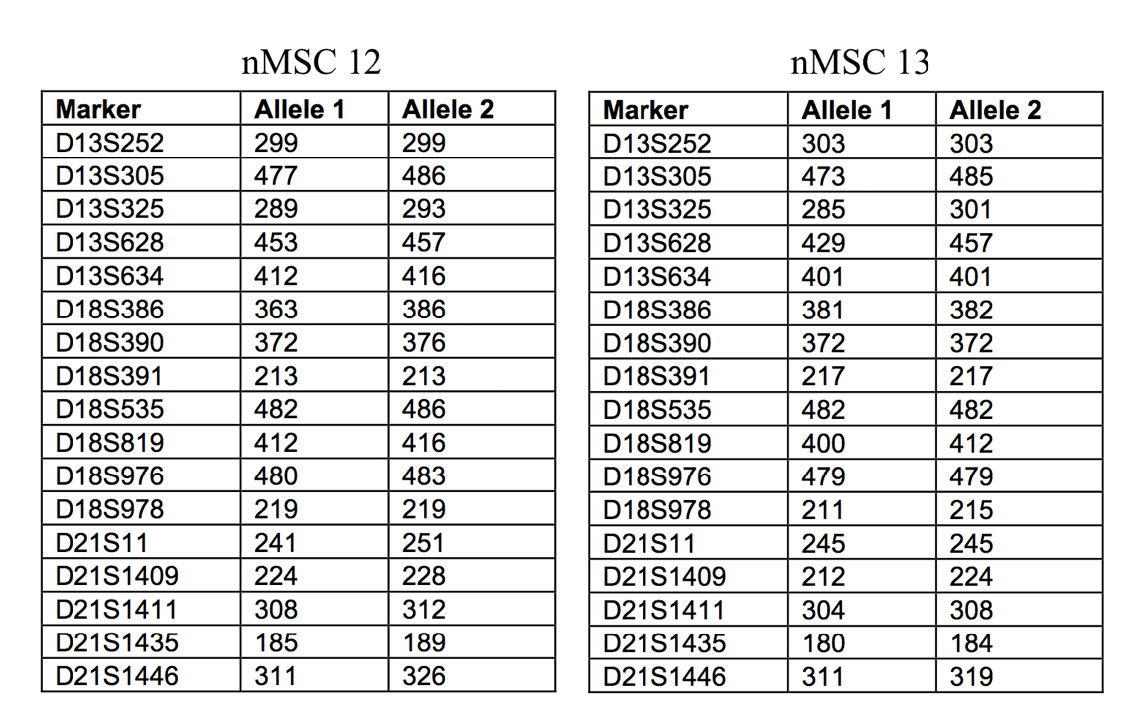


**Figure S2.** Microsatellite markers specific for chromosomes 13, 18, 21, X and Y were amplified. The sex chromosome markers were consistent with a female complement in both lines. The allele sizes in basepairs, for markers on chromosomes 13, 18 and 21 are listed in the table.


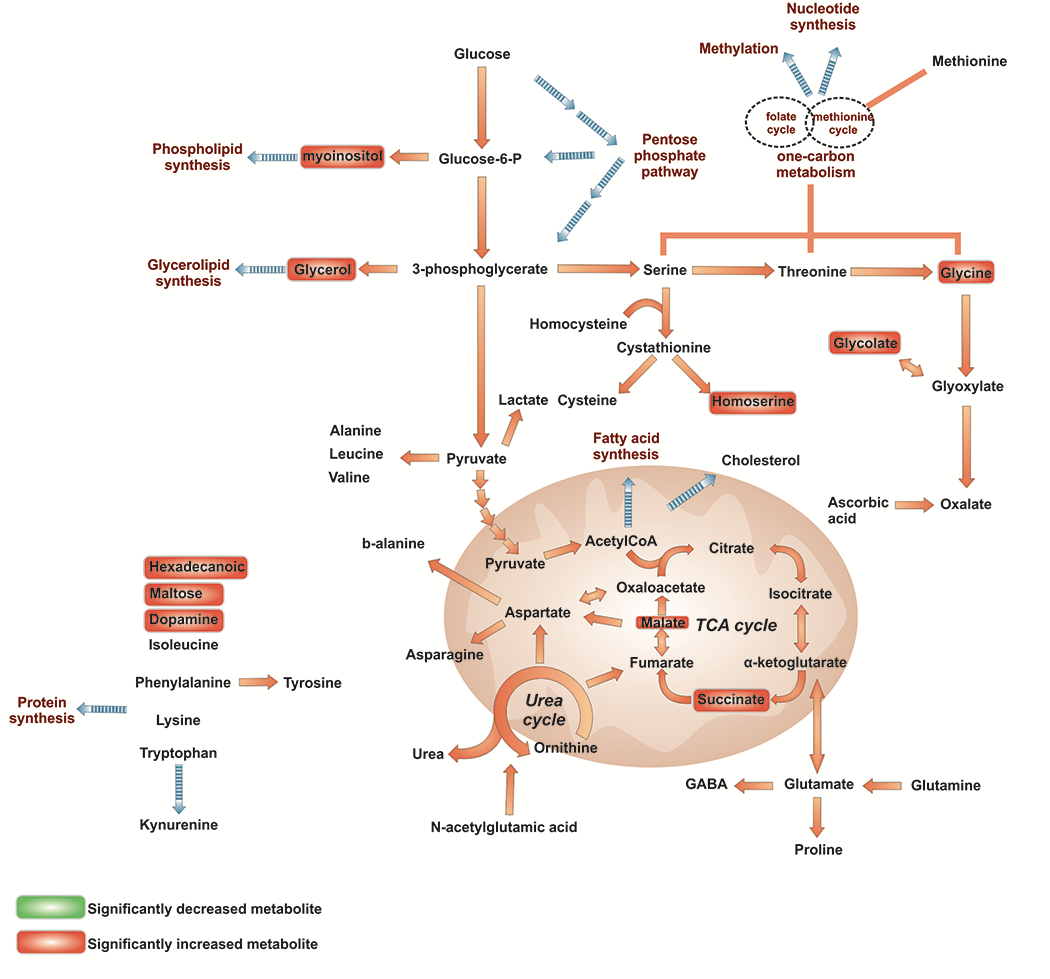


**Figure S3**. IFNG treatment (T) causes significantly increased metabolism in general when compared nMSC (T) with non-treated nMSC.

**Table S1.** Primers used in the study

| **Gene** | **Primers** |
| --- | --- |
| Sendai virus (SeV) | Forward: 5’-GGATCACTAGGTGATATCGAGC-3’  Reverse: 5’-ACCAGACAAGAGTTTAAGAGATATGTATC-3’ |
| β**-**actin (*ACTB*) | Forward: 5’-CAACCGCGAGAAGATGAC-3’  Reverse: 5’-AGGAAGGCTGGAAGAGTG-3’ |
| *FABP4* | Forward: 5’-AGCACCATAACCTTAGATGGG-3’  Reverse: 5’-CGTGGAAGTGACGCCTTTCA-3’ |
| *COL11A1* | Forward: 5’-CCAGCGTCTGTTGGTTCAGT-3’  Reverse: 5’-CAGCTTCCCCTTTCTCTCCT-3’ |
| *RUNX2* | Forward: 5’-TTACTTACACCCCGCCAGTC-3’  Reverse: 5’-TATGGAGTGCTGCTGGTCTG-3’ |
| *TBP* | Forward: 5’-CAGCGTGACTGTGAGTTGCT-3’  Reverse: 5’-TGGTTCATGGGGAAAAACAT-3’ |
| *GUSB* | Forward: 5’-AAACGATTGCAGGGTTTCAC-3’  Reverse: 5’-CTCTCGTCGGTGACTGTTCA-3’ |
| *HPRT1* | Hs02800695_m1 (Thermo Fisher) |
| *IDO1* | Hs00984148_m1 (Thermo Fisher) |
| *CXCL11* | Hs04187682_g1 (Thermo Fisher) |
| *GBP4* | Hs00925073_m1 (Thermo Fisher) |

**Table S2.** MSCA-1+ subpopulation in primary WJ MSC cultures from 7 different donors.

| **Donor #** | **8** | **9** | **10** | **11** | **12** | **13** | **14** |
| --- | --- | --- | --- | --- | --- | --- | --- |
| **MSCA-1+ cells [%]** | 15.8 | 4.4 | 23.5 | 13.5 | 38.8 | 86.1 | 29.5 |

Red: two lines with highest expression of MSCA-1, nMSC from donor 12 and 13, were selected for the future experiments.
